# Encoding of Luminance and Contrast by Linear and Nonlinear Synapses in the Retina

**DOI:** 10.1016/j.neuron.2011.12.023

**Published:** 2012-02-23

**Authors:** Benjamin Odermatt, Anton Nikolaev, Leon Lagnado

**Affiliations:** 1 MRC Laboratory of Molecular Biology, Hills Road, Cambridge CB2 0QH, UK

## Abstract

Understanding how neural circuits transmit information is technically challenging because the neural code is contained in the activity of large numbers of neurons and synapses. Here, we use genetically encoded reporters to image synaptic transmission across a population of sensory neurons—bipolar cells in the retina of live zebrafish. We demonstrate that the luminance sensitivities of these synapses varies over 10^4^ with a log-normal distribution. About half the synapses made by ON and OFF cells alter their polarity of transmission as a function of luminance to generate a triphasic tuning curve with distinct maxima and minima. These nonlinear synapses signal temporal contrast with greater sensitivity than linear ones. Triphasic tuning curves increase the dynamic range over which bipolar cells signal light and improve the efficiency with which luminance information is transmitted. The most efficient synapses signaled luminance using just 1 synaptic vesicle per second per distinguishable gray level.

## Highlights

► Bipolar cell synapses display luminance sensitivities varying over 10^4^ ► Transmission from ON and OFF cells can switch polarity ► Nonlinear synapses signal luminance more efficiently than linear ones ► Nonlinear synapses display higher contrast-sensitivity than linear ones

## Teaser

How does the visual system extract information from visual scenes with wide variations in luminance? Odermatt et al. found that bipolar cells have varying sensitivity to luminance and extend the range of luminances that they signal by generating responses with different polarities.

## Introduction

The neural code transmitting information in the nervous system is contained in the electrical activity of large numbers of neurons and the secretory activity of many more synapses (Dayan and Abbott, 2005). Understanding these codes is a formidable experimental challenge. Most population measurements of signals in circuits have focused on somatic spikes, monitored directly using electrophysiology or indirectly using optical techniques. But the generation of spikes is determined by a much more numerous, diverse, and plastic component of neural circuits—synapses (Abbott and Regehr, 2004). How is information encoded across a population of synapses?

Sensory systems provide an excellent context in which to study neural codes because the experimenter has control over the information to be represented. An intensively studied example is the retina, where a multielectrode array can be used to record spiking activity across the population of ganglion cells that deliver the results of visual processing to the brain (Meister et al., 1995; Baccus, 2007; Gollisch and Meister, 2010). But we still have only a rudimentary understanding of how this output is generated by neurons and synapses within the retina. Take, for example, the most basic statistic of a visual stimulus—the distribution of intensities (or luminances) that it contains. Highlights and shadows within visual scenes can differ in intensity by 4–5 log units (Rieke and Rudd, 2009; Pouli et al., 2010), and the visual system of primates senses luminance over a similar range (Ueno et al., 2004; Hamilton et al., 2007). Yet during the day, light is converted into neural signals through an array of cone photoreceptors with a dynamic range of only ∼10^2^ and with uniform sensitivity to light (Naka and Rushton, 1966a; Normann and Perlman, 1979; Schnapf et al., 1990). This discrepancy raises two basic questions. How is the dynamic range of luminance signaling increased after light has been converted into an electrical signal? And, more broadly, how is information about luminance encoded downstream of photoreceptors?

To investigate these questions we have used fluorescent proteins that report synaptic activity. We focus on the second stage of processing in the retina, where bipolar cells in the inner plexiform layer (IPL) transmit to ganglion cells (Baccus, 2007; Masland, 2001). To allow these measurements to be made in vivo across the whole population of bipolar cells, we generated zebrafish expressing sypHy—a fluorescent protein that reports synaptic vesicle fusion (Granseth et al., 2006). Additionally, we monitored the presynaptic calcium signal driving neurotransmission using SyGCaMP2 (Dreosti et al., 2009, 2011). We find that luminance information is transferred to the inner retina using synapses that are tuned to intensities varying over 4–5 log units. Strikingly, half the synapses in the ON and OFF pathways signaled luminance through a triphasic intensity-response function with a distinct minimum and maximum. Using ideal observer analysis (Smith and Dhingra, 2009; Geisler, 2011), we find that this tuning curve doubles the efficiency with which individual synapses use vesicles to signal luminance and also increases their sensitivity to temporal fluctuations in intensity (i.e., contrast). These results demonstrate how the population of bipolar cell synapses uses a combination of strategies to transfer information about the luminance and contrast of a visual stimulus.

## Results

### The *Ribeye A* Promoter Targets Expression to Ribbon Synapses

**Figure 1:**
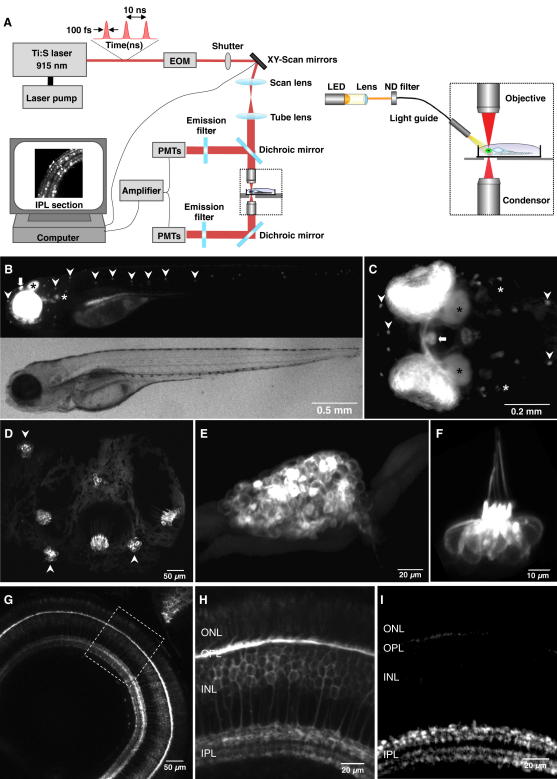
The Zebrafish *ribeye a* (*ctbp2*) Promoter Drives Expression in Neurons Containing Ribbon Synapses (A) Imaging synaptic reporters in the retina of live zebrafish using a two-photon microscope. Full-field stimuli were applied through a light guide. (B and C) A stable transgenic fish expressing membrane targeted EGFP (memEGFP) under control of 1.8 kb of the genomic sequence upstream of the *ribeye a* gene (*Tg(−1.8ctbp2:memEGFP)lmb*). At 4 dpf, all sensory organs known to express ribbon synapses were labeled, including the retina, the inner ear (white asterisk), the pineal gland (bold arrow), and the neuromasts (arrow heads). (B) Side view, (C) top view of the fish head. Additionally EGFP expression can be seen in the optic nerve and the optic tectum (black asterisk). (D) In a fish at 7 dpf, EGFP expression is driven in hair cells of the inner ear (side view) and maculae (not shown). (E and F) EGFP expression in the pineal gland and a neuromast, respectively (side view; 7 dpf). (G) In the retina, the *ribeye a* promoter drove expression of memEGFP in photoreceptors and bipolar cells. (H) Labeled photoreceptors in the outer nuclear layer (ONL), their terminals in the outer plexiform layer (OPL), cell bodies of bipolar cells in the inner nuclear layer (INL), and their terminals in the inner plexiform layer (IPL). (I) Expression of sypHy localized to terminals in the OPL and IPL in the stable *Tg(−1.8ctbp2:sypHy)lmb* line used in this study (see also Figure S1).

Transmission of the visual signal to the inner retina was imaged in live zebrafish by targeting sypHy and SyGCaMP2 to ribbon synapses of bipolar cells (Figure 1A). To target expression of these reporters to retinal bipolar cells we cloned the promoter of the *ribeye a* gene (Wan et al., 2005). Ribeye is the major structural protein of the presynaptic ribbon that holds vesicles close to the active zone (Schmitz et al., 2000). In zebrafish, there are two *ribeye* genes, a and b, but only a is expressed in retinal bipolar cells. Figures 1B–1H show the expression of a membrane-fused (mem)EGFP driven by 1.8 kb of the promoter region upstream of the *ribeye a* ATG. Robust expression was obtained in all ribbon synapses in the eye, vestibular organ, lateral line, and pineal. In the retina, expression of sypHy under the *ribeye a* promoter was localized to the pedicles of cones in the OPL, and the synaptic terminals of bipolar cells distributed through all layers of the IPL (Figure 1I). Expression of sypHy was strong both in bipolar cells expressing PKC-α, which are generally thought to be ON, and those negative for PKC-α, generally thought to be OFF (Figure S1). Thus, the *ribeye a* promoter efficiently drove expression across the complete population of bipolar cells in the zebrafish retina.

### In Vivo Imaging of Synaptic Transmission across a Population of Sensory Neurons

**Figure 2:**
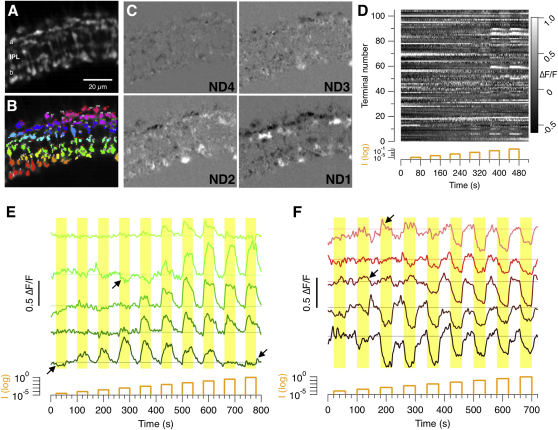
In Vivo Imaging of Synaptic Transmission in the Retina (A) Field of view showing sypHy expression in synaptic terminals of bipolar cells in the IPL of a fish at 10 dpf. (B) ROIs from the same field highlighted in different colors. When viewed at highest resolution, numbers mark ON terminals and red numbers OFF. Nonresponding terminals numbered in white. (C) Difference images highlighting the change in sypHy fluorescence in response to steps of light. Attenuation of the light source is shown in log units (ND 4 to ND 1). Darker areas show OFF terminals; brighter areas are ON. (D) Raster plot showing the relative change in fluorescence (ΔF/F) for each ROIs marked in (B). The intensity of the stimulus was increased in steps of 1 log unit, with a maximum intensity of 5.5 × 10^5^ photons/μm^2^/s. (E) Responses of five individual ON terminals to light steps increasing in intensity by 0.5 log units. Darker hues indicate more sensitive terminals. The black arrows highlight some examples of switches in response polarity. (F) Responses of five individual OFF terminals (see also Figure S2).

A view of the IPL in which more than 100 terminals could be distinguished is shown in Figures 2A–2C, together with the change in sypHy fluorescence generated by four presentations of full-field amber light, each step increasing in intensity by a factor of 10 (see also Movie S1 available online). ON terminals became brighter in response to light, reflecting the acceleration of vesicle fusion, while OFF terminals became dimmer, reflecting a slowing down of vesicle release and a net removal of pHluorin from the surface by endocytosis (Lagnado et al., 1996). The relative change in fluorescence over time for all these 100 terminals is shown in the raster plot in Figure 2D. Some synapses generated a response to the infrared laser at the beginning of an imaging episode, but in most cases this response was small and complete within 5–10 s (Figure S2). A strength of this approach is that signal transfer could be monitored across hundreds of bipolar cell terminals simultaneously, through all layers of the inner retina. The spatial resolution was not, however, sufficient to monitor signals at individual active zones within these terminals.

Examples of sypHy signals from individual ON and OFF terminals are shown in Figures 2E and 2F, measured in response to a series of light steps increasing in intensity by 0.5 log units. These traces illustrate three unexpected properties of signal transmission that we analyze in this paper. First, individual terminals exhibited a striking variability in their sensitivity to light. Second, in some terminals, the relation between response amplitude and light intensity was not monotonic, but passed through a maximum. Third, in some terminals the response to a dim light was of the opposite polarity to that of a brighter light (arrowed in Figures 2E and 2F).

**Figure 3:**
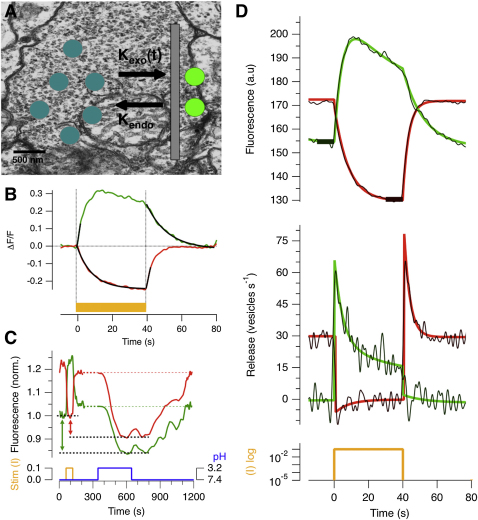
Calculating the Rate of Vesicle Release from sypHy Signals (A) Electron micrographs indicate that there are about 15,000 vesicles in an average bipolar cell terminal (background). The number of unquenched sypHy molecules on the surface depends on both the rate of exocytosis and the rate of endocytosis (foreground). (B) Estimating the rate of endocytosis in vivo: comparison of the sypHy signal in response to a bright step of light (ND 1) averaged from a population of 95 ON terminals (green) and 272 OFF terminals (red). In OFF terminals, the sypHy signal decayed exponentially with τ ∼10 s (black line). In ON terminals, the signal decayed at the same rate when the light step was turned off. In both channels, acceleration of vesicle release generated a sypHy signal that rose at a constant average rate for the first 2 s (black lines). (C) Estimation of the sypHy surface fraction (α_min_) by acid quenching. First, responses to a step of bright light (ND 1) were measured in ON and OFF terminals at pH 7.4. Then, sypHy molecules on the surface in darkness were quenched with a solution at pH 3.2. The difference between the minimum fluorescence at pH 7.4 and pH 3.2 reflects quenching of the surface fraction (dashed lines). Traces averaged from 10 fish. α_min_ averaged 0.8% in ON and OFF terminals (see also Figure S3). (D) Upper traces: average fluorescence response of ON (green) and OFF (red) terminals to a 40 s light step (ND 1). The response and recovery phases could both be described as double-exponential functions (smooth lines). Lower traces: a comparison is shown of the conversion of the fluorescence response to rates of vesicle release, V_exo_(t), using the raw sypHy signal (noisy trace) and the fitted traces that minimize noise. Thick black bars in upper graph show the values of F_min_ used for this calculation, as described in Experimental Procedures.

To investigate the transmission of luminance signals quantitatively, we calculated the rate of vesicle release taking into account the fact that sypHy signals are dependent on both exocytosis, occurring with a variable rate k_exo_(t), and endocytosis, occurring with rate-constant k_endo_ (Figure 3A). The absolute release rate at any time point, V_exo_(t), was calculated as:

(Equation 1) $$V_{exo}\left(t\right)=a\left[\frac{dF}{dt}+\left(k_{endo}*\left(F\left(t\right)-b\right)\right)\right]$$

where F(t) is the actual total fluorescence measured over the terminal, and *a* and *b* are constants dependent on the total number of vesicles in the terminal and the fraction of these that are unquenched on the surface. The derivation of this relation is described in the Experimental Procedures. The rate constant k_endo_ has been measured in isolated bipolar cells using the capacitance technique and is ∼0.1 s^−1^ during maintained activity (von Gersdorff and Matthews, 1994; Neves and Lagnado, 1999). We found that k_endo_ was also ∼0.1 s^−1^ in vivo, as measured from the decline in the sypHy signal when exocytosis was minimized (Figure 3B). Calculation of constants *a* and *b* required the following: the cross-sectional area of the terminal within an optical section ∼2 μm thick (obtained by underfilling the back aperture of the objective); the average density of vesicles in a bipolar cell terminal, which was estimated as ∼1,050 per μm^3^ from electron micrographs (Figure 3A), and an estimate of the sypHy surface fraction (α_min_), which was measured by acid quenching the pHluorin on the surface membrane (Figures 3C and S3 and Experimental Procedures).

The dynamic range of signaling through ON and OFF channels was similar. Switching on a bright light from a dark-adapted state accelerated vesicle release to an average peak rate of ∼65 vesicles s^−1^ in ON terminals, while switching this light off accelerated release to ∼75 vesicles s^−1^ in OFF terminals (Figure 3D). Terminals of bipolar cells in zebrafish contain an average of about 6 ribbons (unpublished observations), so these measurements converts to release rates of ∼12 vesicles s^−1^ per synaptic contact. These estimates are similar to measurements of the transient component of exocytosis from ON bipolar cells estimated by analysis of noise in postsynaptic ganglion cells (∼17 vesicles s^−1^ per contact; Freed, 2000b). Notably, the synaptic output from both ON and OFF terminals recovered partially from these peak rates with time constants of ∼3–7 s (Figure 3D), reflecting adaptation to luminance.

### Variations in Luminance Sensitivity across a Population of Synaptic Terminals

**Figure 4:**
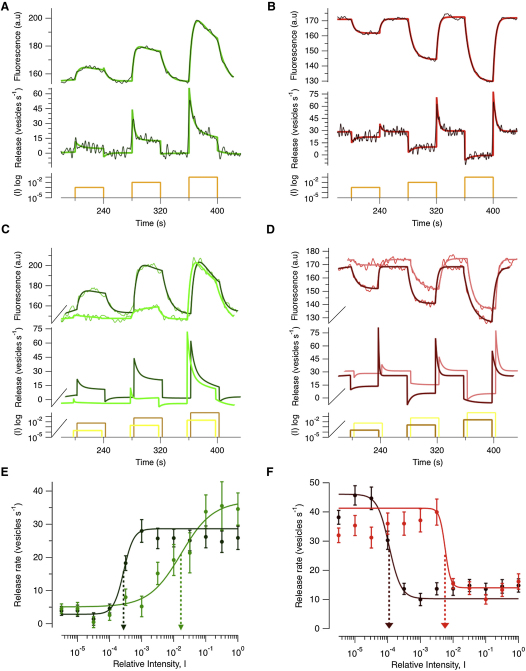
Variations in the Intensity-Response Relation of Different Synaptic Terminals (A) Upper trace, average fluorescence response of ON terminals to light steps of three different intensities. Smooth lines show the description of these responses by a series of double exponential fits. Lower trace, conversion of the upper signal to rates of vesicle release using both original and fitted traces. (B) The rate of vesicle release in OFF terminals calculated in the same way. (C) Averaged response from the 20% most sensitive ON terminals (dark green) and the 20% least sensitive (light green). (D) Averaged response from the 20% most sensitive OFF terminals (dark red) and the 20% least sensitive (light red). (E) Peak release rate at light onset as a function of the relative intensity could be described using a Hill function. Dark green: averages of the 33% most sensitive ON terminals (n = 35, I_1/2_ = 2.7 × 10^−4^, h = 2.8). Light green: 33% least sensitive terminals (n = 37, I_1/2_ = 1.7 × 10^−2^, h = 0.9). Dashed arrows show I_1/2_. Error bars are SEM. (F) Hill function fit to the intensity-response relation of two subsets of OFF terminals: the 33% most sensitive (dark red; n = 65, I_1/2_ = 1.1 × 10^−4^, h = 2.4) and 33% least sensitive (light red; n = 58, I_1/2_ = 5.7 × 10^−3^, h = 5.1).

The conversion of sypHy signals to rates of vesicle release is shown for light steps of three different intensities in Figure 4A (ON terminals) and Figure 4B (OFF). These records were obtained by averaging over the two populations, irrespective of sensitivity. The variation within each population is illustrated by the individual examples in Figures 2E and 2F and by averaging responses from the 20% of terminals at the two extremes of the sensitivity distribution, as shown in Figures 4C and 4D. For both ON and OFF cells, we only analyzed the initial response at light onset, measured from a dark-adapted state. The intensity-response relations of each of these four subsets of synapses is shown in Figures 4E and 4F. A good description was obtained using the Hill equation:

(Equation 2) $$R=R_{\text{max}}\left(\frac{I^{h}}{I^{h}+I_{1/2}^{h}}\right)$$

where sensitivity is quantified as the intensity producing the half-maximal response (I_1/2_), and the Hill coefficient (h) is the power law describing how the response grows at low intensities.

**Figure 5:**
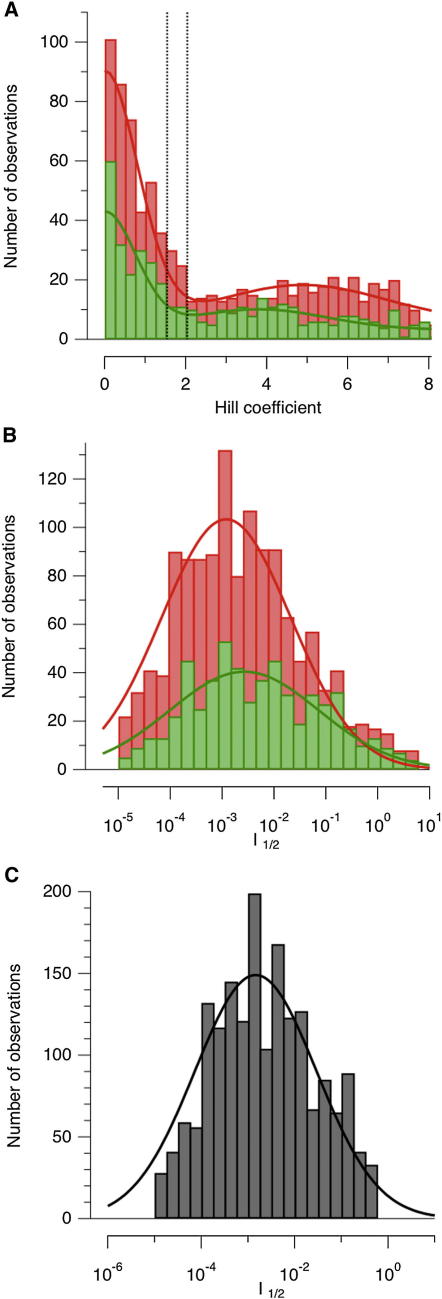
Nonlinearities and Sensitivity across the Population of Bipolar Cell Terminals (A) The distribution of Hill coefficients (h) across individual synaptic terminals. ON (green, n = 536), OFF (red, n = 1,218). Both distributions show a distinct population with h < 1.5 (termed “linear”), and a second population with h > 2.0 (“nonlinear”). The fitted function is the sum of two Gaussians. (B) Distribution of I_1/2_ across a complete sample of ON (green) and OFF (red) terminals from 178 experiments. The fitted curves are log-normal functions with the shape $\exp\left[-{\left(\ln\left(I/I_{0}\right)/2\sigma \right)}^{2}\right]$. (C) Distribution of I_1/2_ across the complete sample of ON and OFF terminals (n = 1,754). I_0_, the peak of the distribution, occurs at a relative intensity of 1.47 ± 0.39 × 10^−3^, equivalent to 8.1 × 10^2^ photons μm^−2^ s^−1^. In comparison, the threshold for activation of cones is about 10^2^ photons μm^−2^ s^−1^ (Schnapf et al., 1990). The width of the distribution $\sqrt{2}\sigma$ was 4.2 ± 0.4 log units. There was no correlation between I_1/2_ and h (see also Figure S4).

For cones, h is ∼1 and I_1/2_ is constant across the whole population when measured at the optimal wavelength (Baylor et al., 1987; Normann and Perlman, 1979). The synaptic output of cones and voltage responses in the soma of bipolar cells also display a Hill coefficient around 1 (Choi et al., 2005; Euler and Masland, 2000). But in synapses of bipolar cells, both h and I_1/2_ varied widely. The distribution of h is shown by the histograms in Figure 5A. Two components can be seen: a sharp peak at h below about 1.5, and a much more widely distributed component at h greater than about 2.0. Supralinearity, which we defined as h > 2, was observed in 66% of OFF and 62% of ON terminals. In other words, some terminals signaled luminance almost in an all-or-none manner. Individual examples of this behavior are shown in Figures 2E (ON) and F (OFF) and Figures S5A and S5B. Thresholding in the synaptic output of bipolar cells is not easily explained by the idea that these are graded neurons that simply respond to linear synaptic inputs and is more likely to reflect active conductances within the synaptic terminals (Burrone and Lagnado, 1997; Baden et al., 2011).

The value of I_1/2_ across the population of bipolar cells varied over 4 log units and the distribution had a characteristic shape for both ON and OFF channels—normal on a log scale (Figures 5B and 5C). Strikingly, a number of studies have found that the distribution of luminance in natural scenes is also log normal (Richards, 1982; Brady and Field, 2000; Geisler, 2008). Although the shape of these distributions appears relatively constant, the width varies: a scene in bright sunlight containing deep shadows might contain luminances varying across 4–5 log units (Pouli et al., 2010; Rieke and Rudd, 2009). The population of bipolar cells can, however, transmit luminance information to the inner retina across synaptic terminals with sensitivities that are distributed widely enough to encode scenes with these high dynamic ranges. The log-normal distribution of sensitivities also suggests that more synapses will be matched to the luminance values most prevalent in the image falling on the retina.

### Triphasic Tuning Curves and Switches in Polarity

**Figure 6:**
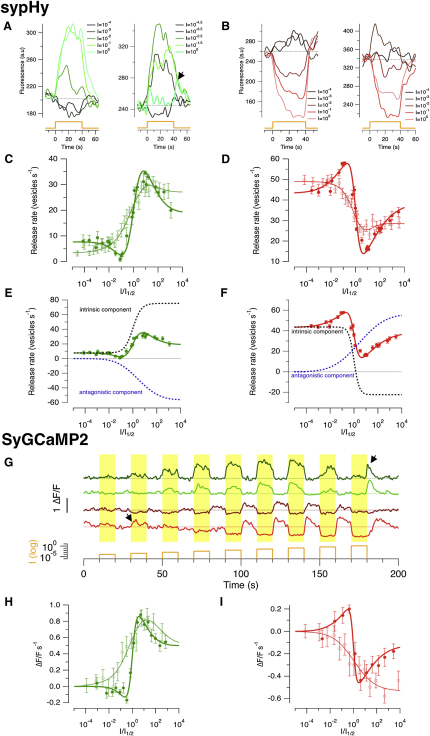
Linear and Nonlinear Tuning Curves Encoding Luminance (A) SypHy responses from two ON terminals elicited by a series of light steps increasing in intensity by one log unit. Lower intensities caused a *decrease* in vesicle release. The right hand example shows suppression of release at the highest intensity, where there is a rebound burst of exocytosis at light offset (i.e., an OFF response; black arrow). (B) Responses from two OFF terminals. Lower intensities caused an *increase* in vesicle release. (C) The average shape of the intensity-response function for ON terminals. Linear (thin line) and nonlinear (bold) were averaged separately after normalizing the relation measured in each terminal to the intensity producing the half-maximal response, I′. The function fitted to both curves is of the form$$V_{exo}=A+Int\left(\frac{I^{\prime h}}{I^{\prime h}+1}\right)+\frac{Antag}{\sigma \sqrt{2\pi }}\int_{0}^{I^{\prime }}\text{exp}\left[-{\left(\frac{\text{ln}\left(I^{\prime }\right)}{2\sigma }\right)}^{2}\right]dI^{\prime }$$where A is an offset, Int is a scaling factor for the “intrinsic” component described by the Hill equation, Antag is the scaling factor for the “antagonistic” component described by the cumulative density function of a log-normal distribution, and $\sqrt{2}\sigma$ is the width of that distribution in log units. For linear synapses, A = 3.4 vesicles s^−1^, Int = 76.1 vesicles s^−1^, h = 0.6, Antag = −52.23, width = 3.11. For nonlinear synapses, A = 7.7 vesicles s^−1^, Int = 67.7 vesicles s^−1^, h = 1.3, Antag = −56.34, width = 3.23. The way the intrinsic and antagonistic components sum to generate the nonlinear tuning curve is shown in (E). (D) The average shape of the intensity-response function for OFF terminals at light onset. For linear synapses (thin line), A = 49 vesicles s^−1^, Int = −57.8 vesicles s^−1^, h = 0.85, Antag = 37.3, width = 2.1. For nonlinear synapses (bold line), A = 43.3 vesicles s^−1^, Int = −64.3 vesicles s^−1^, h = 2.11, Antag = −59.2, width = 4.46. The summation of the intrinsic and antagonistic components to generate the nonlinear tuning curve is shown in (F). (G) A comparison with luminance tuning assessed using SyGCaMP2 to monitor the presynaptic calcium signal. Each trace shows responses of individual terminals. Arrows point to examples of switches in the polarity of the response to dim or bright lights (see also Figure S5). The average shape of the intensity-response function are shown in (H) for ON terminals and (I) for OFF. The response was quantified as the initial rate of change of the SyGCaMP2 signal relative to fluorescence in the dark. Linear (thin line) and nonlinear (bold line) terminals were distinguished in the same way as for sypHy measurements, according to the Hill coefficient best describing the major part of the response. The luminance tuning curves are very similar to those measured using sypHy (C and D). All error bars are SEM.

The tuning curve of a sensory neuron is a key determinant of the information that it can transmit about a stimulus. Several theoretical studies have suggested that sharper tuning curves within individual neurons can improve the overall efficiency of population codes, in part because the finest discrimination occurs over the range of stimulus strengths that most rapidly alter the neurons response (Brunel and Nadal, 1998; Pouget et al., 1999; Seriès et al., 2004; Butts and Goldman, 2006). Tuning curves similar to Hill functions or Gaussians can only provide this advantage at the cost of signaling over a narrower range of stimulus strengths, but we found a subset of bipolar cell synapses in which the dynamic range of signaling was increased by an unexpected mechanism: switching the polarity of the exocytic response as a function of luminance. Examples of sypHy signals from such terminals are shown in Figure 6A (ON) and Figure 6B (OFF): the response to a dim light was of the opposite polarity to the larger response to a brighter light.

We examined the tuning curves of linear and nonlinear synapses more closely by normalizing the relation measured in individual terminals to I_1/2_ and then averaging within the linear and nonlinear classes (Euler and Masland, 2000). The response of nonlinear ON synapses did not saturate as light intensity increased but passed through a minimum (transition from phase one to two) and then a maximum (transition from phase two to three) before reaching a steady state (Figure 6C). The response of nonlinear OFF synapses was roughly an inversion of this triphasic shape (Figure 6D). A good empirical description of triphasic tuning curves could be obtained by considering them as the sum of two components, which we termed “intrinsic” (black traces in Figures 6E and 6F), and “antagonistic” (blue traces). The expression fitted to these curves is

(Equation 3) $$V_{exo}=A+Int\left(\frac{I^{\prime h}}{I^{\prime h}+1}\right)+\frac{Antag}{\sigma \sqrt{2\pi }}\int_{0}^{I^{\prime }}\text{exp}\left[-{\left(\frac{\text{ln}\left(I^{\prime }\right)}{2\sigma }\right)}^{2}\right]dI^{\prime }$$

where I′ is the intensity normalized to I_1/2_, A is an offset, Int is a scaling factor for the “intrinsic” component described by a Hill function, Antag is the scaling factor for the “antagonistic” component, described by the cumulative density function of a log-normal distribution, and $\sqrt{2}\sigma$ is the width of that distribution in log units. The value of σ varied between 3.0 and 4.5 log units and was therefore similar to the distribution of sensitivities across the population of terminals shown in Figure 5C. The growth of the antagonistic component in parallel with the number of bipolar cells activated suggests that this signal may originate from neighboring bipolar cells that are progressively recruited as the light intensity increases. After normalization and averaging, a much weaker antagonistic component could also be detected in the “linear” group of synaptic terminals (Figures 6A and 6B, thin fitted traces).

We are not aware that tuning functions with a triphasic form have been described before in a sensory neuron. A switch in the polarity of the synaptic output of bipolar cells is especially surprising because the electrical response in the soma is determined by the type of glutamate receptor sensing transmitter release from photoreceptors: a metabotropic receptor in ON cells and an ionotropic receptor in OFFs (Masland, 2001). We therefore investigated synaptic tuning curves in bipolar cells by imaging a second variable reflecting signal transmission—the calcium signal driving neurotransmitter release. These experiments were carried out using a line of transgenic zebrafish expressing SyGCaMP2 (Dreosti et al., 2009). Use of the *ribeye* promoter described in Figure 1 allowed us to localize expression of SyGCaMP2 to ribbon synapses. Figure 6G shows examples of responses from individual ON and OFF bipolar cell terminals stimulated with steps of light over the same intensity range used in experiments employing sypHy. The top two traces provide examples of sustained ON cells that generate transient OFF responses at the highest luminance tested (arrowed); the next trace is an OFF cell in which the tuning curve passes through a maximum, and the bottom trace is an example of an OFF cell that generates ON responses at the lowest intensities (arrowed).

Collected results using SyGCaMP2 are shown in Figures 6H and 6I and are expanded on in Figures S4, S5C, and S5D (using 100 ON synaptic terminals and 39 OFF). These tuning curves were constructed using the same general approach applied to sypHy measurements, except that the response was quantified as the initial rate of change of SyGCaMP2 fluorescence normalized to the baseline. The tuning curves of linear (49%) and nonlinear (51%) terminals were described well by Equation 3, with shape parameters σ and h very similar to those estimated by assessing the exocytic response using sypHy (cf. Figures 6C and 6D).

### Nonlinear Synapses Transmit Luminance Information More Efficiently

How do the “linear” and “nonlinear” tuning curves affect the encoding of a sensory stimulus? A useful way to frame this question is to ask how many different levels of luminance (N_L_) might be discriminated by observing the output of the bipolar cell terminal, taking into account the variability inherent in the process of synaptic transmission (Jackman et al., 2009; Smith and Dhingra, 2009). At many synapses, including ribbon synapses of bipolar cells, vesicle release follows Poisson statistics, with a variance equal to the mean (Katz and Miledi, 1972; Laughlin, 1989; Freed, 2000a, 2000b). The discriminability, d′, of two stimulus values differing by δs will depend on the signal-to-noise ratio (SNR) (Geisler, 2011) as

(Equation 4) $$d^{\prime }=\sqrt{SNR}$$

A convenient way to calculate *d′* across the whole tuning curve is from the Fisher Information, I_F_, a quantity that places a limit on the best estimate of a stimulus that can be extracted from the response of a neuron using any unbiased decoding scheme (Dayan and Abbott, 2005). If the response varies according to Poisson statistics, I_F_ can be calculated from the derivative of the tuning curve f(s):

(Equation 5) $$I_{F}=\frac{{\left[d\left(f\left(s\right)\right)/ds\right]}^{2}}{f\left(s\right)}$$

and

(Equation 6) $$d^{\prime }=\delta s\sqrt{I_{F}\left(s\right)}.$$

The overall performance of the neuron can then be quantified by integrating d′ over s to estimate the number of different stimulus values that can be resolved (Barlow et al., 1987; Smith and Dhingra, 2009):

(Equation 7) $$N_{L}=\int_{0}^{\infty }\sqrt{\frac{f^{\prime }{\left(s\right)}^{2}}{f\left(s\right)}}ds.$$

**Figure 7:**
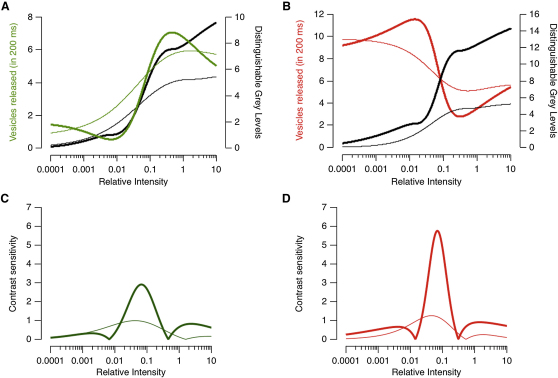
Nonlinear Tuning Curves Improve Performance (A) The tuning curves of ON terminals showing the average number of vesicles released over a 0.2 s time window (green line, left axis), and the number of gray levels distinguishable by counting vesicles over this interval using an ideal observer model (black line, right axis). Thin lines are the linear synapses, and bold lines nonlinear. (B) A similar comparison for OFF terminals. (C and D) Predicted contrast sensitivity functions derived from the luminance tuning curves of ON terminals in (A) and OFF terminals in (B).

We used this approach to calculate the number of changes in luminance (N_L_) or gray levels that could be distinguished from the synaptic output if vesicles were counted over a time window of 200 ms, roughly equivalent to the integration time of a bipolar cell (Ashmore and Falk, 1980). A given rate of vesicle release did not necessarily map onto a single luminance value because tuning curves were not monotonic, but this does not invalidate the approach for estimating the number of distinguishable gray levels because the calculation is based on discriminating one level of luminance from another rather than estimating the absolute value (Barlow et al., 1987). On average, a single linear ON terminal distinguished ∼5.5 gray levels, while a nonlinear terminal distinguished ∼10 (Figure 7A). In the OFF channel, a single linear terminal distinguished ∼5.5 gray levels, while a nonlinear terminal distinguished ∼14 (Figure 7B). Thus, nonlinear synapses were capable of detecting 2 to 3 times as many gray levels as the linear class.

Discriminability can always be improved by counting more vesicles, for instance by increasing the release rate. But in practice the design of neural circuits is constrained by the need to encode and transmit information in an energy-efficient manner (Attwell and Gibb, 2005; Laughlin, 2001). The retina devotes considerable resources to transmitting the visual signal to the IPL: synaptic terminals of bipolar cells occupy a sizeable fraction of the retinal volume (Figure 1H) and contain large numbers of vesicles and mitochondria. How efficiently do different bipolar cells use these resources to encode luminance? To investigate this question, we quantified the cost of signaling luminance by dividing the average rate of vesicle release, $\langle V_{exo}\rangle$, during normal activity by the total number of distinguishable gray levels (N_L_).

(Equation 8) $$Cost=\frac{\langle V_{exo}\rangle }{NL}$$

To calculate $\langle V_{exo}\rangle$, we assumed that bipolar cells randomly sample a log-normal distribution of luminances mirroring the distribution of sensitivities in Figure 5C. If the probability density function of luminance is f(I),

(Equation 9) $$\langle V_{exo}\rangle =\langle V_{exo}\left(I\right)\times f\left(I\right)\rangle$$

The mean rate of vesicle release through linear ON terminals was 15.5 vesicles s^−1^, so the average cost of encoding luminance was 2.51 vesicle s^−1^ per gray level in an observation time of 200 ms. Nonlinear ON terminals operated at an average cost of 1.09 vesicle s^−1^ per gray level distinguished, demonstrating that the improvement in performance did not come at the expense of more vesicles (Figure 7A). In the OFF channel, nonlinear synapses were 2.5 times as efficient as linear ones.

### Nonlinear Synapses Are More Sensitive to Contrast

Although some ganglion cells primarily signal the mean luminance of a stimulus, many more also respond to fluctuations in intensity around this mean (contrast) (Baccus, 2007; Demb, 2008; Masland, 2005). To investigate how the luminance tuning curves of bipolar cell synapses affected the signaling of temporal contrast we began with an analysis based on an ideal observer model, in a manner similar to Choi et al. (2005). If vesicles are released according to Poisson statistics, a change in luminance from s_1_ to s_2_ will be detected with SNR:

(Equation 10) $$SNR=\frac{f\left(s_{1}\right)-f\left(s_{2}\right)}{\sqrt{f\left(s_{1}\right)+f\left(s_{2}\right)}}$$

From the tuning curves in Figures 7A and 7B, we calculated for each value of s_1_ the nearest value of s_2_ generating a response detectable with a SNR ≥ 1. This threshold contrast will be |(s_1_ – s_2_)|/s_1_, and the contrast sensitivity will be the inverse of this value. Figure 7C plots the average contrast sensitivity of linear and nonlinear ON terminals as a function of the mean luminance, s_1_. Increments and decrements in light intensity are detected with different sensitivities, but for simplicity Figure 7C plots the maximum of the two measures. Three general predictions can be made. First, contrast sensitivity will be strongly dependent on the mean luminance at which it is measured, and will be at a maximum when the luminance tuning curve is steepest i.e., at I_1/2_ (cf. Figure 7A). Second, nonlinear terminals will display a higher maximum contrast sensitivity than the linear class, again because their luminance tuning curves are steeper. A third prediction can be made by comparing the calculated contrast sensitivities of ON terminals (Figure 7C) with OFFs (Figure 7D): OFF terminals will, on average, be more sensitive to contrast than ON terminals.

**Figure 8:**
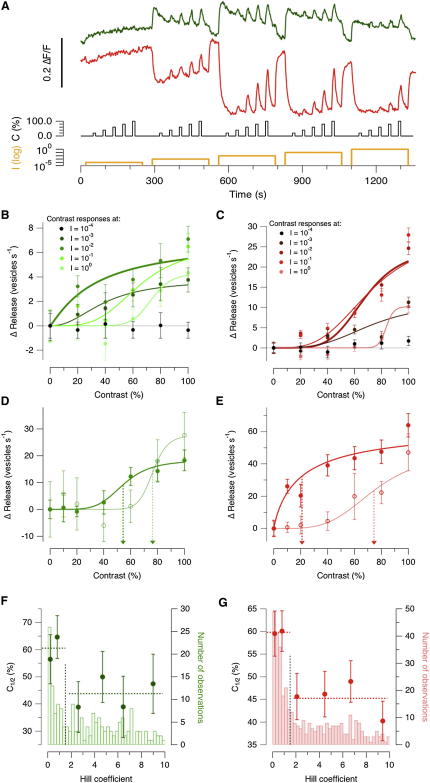
Nonlinear Synapses Display Higher Contrast Sensitivity (A) Assessing contrast sensitivity at different mean luminances. Contrast steps varied between 20% and 100% (5 Hz square wave) and mean luminances increased in steps of one log unit. Traces averaged from 360 ON terminals and 450 OFF. (B) Average contrast-response functions of ON terminals, measured at different mean luminances. Curves are fits of the Hill equation with values of C_1/2_ as follows: I = 10^−3^, C_1/2_ = 39 ± 13%; I = 10^−2^ (bold trace), C_1/2_ = 34% ± 10%; I = 10^−1^, C_1/2_ = 64% ± 7%; I = 10^0^, C_1/2_ = 74% ± 8%. (C) Average contrast-response functions of OFF terminals, measured at different mean luminances. Curves are fits of the Hill equation with the following parameters: I = 10^−3^, C_1/2_ = 72% ± 6%; I = 10^−2^ (bold trace), C_1/2_ = 68% ± 2%; I = 10^−1^, C_1/2_ = 71% ± 3%; I = 10^0^, C_1/2_ = 83% ± 17%. (D and E) Average contrast-response functions in linear and nonlinear terminals. For each individual terminal, contrast steps were applied at the mean light intensity closest to I_1/2_. (D) ON terminals. Fits of the Hill equation with C_1/2_ = 76% ± 8% linear (thin line) and C_1/2_ = 54% ± 7% (nonlinear, bold line). (E) OFF terminals. Fits of the Hill equation with C_1/2_ = 75% ± 9% (linear, thin line) and C_1/2_ = 20% ± 4% (nonlinear, bold line). (F) The relation between C_1/2_ and the Hill coefficient describing luminance tuning in ON terminals (n = 560). These points are superimposed on the distribution of Hill coefficients describing luminance tuning in the same population of terminals (cf. Figure 5A). C_1/2_ was systematically lower in nonlinear terminals. (G) A similar comparison for OFF terminals (n = 890). C_1/2_ was systematically lower in nonlinear terminals. Error bars are SEM (see also Figure S6).

These three predictions were tested experimentally and were all found to hold. By imaging sypHy, the initial exocytic response was measured at contrasts varying between 10% and 100% (5 Hz square wave; Figure S6A). Each stimulus was applied from a steady background, which was varied over 4 log units, as shown by the protocol illustrated in Figure 8A. The contrast-response relations averaged over all ON terminals are shown in Figure 8B, where they are described by fits to the Hill equation. Analogous measurements in OFF cells are shown in Figure 8C. At the lowest mean intensities (I = 10^−4^), there was little response to contrast, indicating that modulation of intensity did not alter the average rate of vesicle fusion. At higher mean intensities (I = 10^−2^ to 10^−3^), the average contrast sensitivity of the population of synapses was significantly higher, reflecting the larger number of terminals tuned to these luminances (Figure 5B). As expected, the average contrast sensitivity falls again at the highest mean intensities (I = 10^0^), reflecting the smaller number of terminals tuned to these luminances. The correlation between the average contrast sensitivity of bipolar cell synapses and the distribution of luminance sensitivities I_1/2_ is also shown in Figure S6B. This correlation can be understood in terms of the results in Figure 7: an individual terminal is expected to exhibit its maximal contrast sensitivity at I_1/2_, so contrast sensitivity averaged across the whole population should parallel the distribution of I_1/2_ (Figure 5C).

To compare the contrast sensitivities of linear and nonlinear terminals, we made measurements at five different mean luminances spanning 4 log units (Figure 8A). However, for each terminal we only used responses to contrast measured at a mean luminance closest to its own value of I_1/2_. In ON terminals, the contrast generating the half-maximal response, C_1/2_, was 76% ± 8% in the linear group, and 54% ± 7% in the nonlinear group (Figure 8D). In OFF terminals, C_1/2_ was 75% ± 9% in the linear group, and 20% ± 4% in the nonlinear (Figure 8E). Thus, nonlinear OFF terminals were the most sensitive to temporal contrast. The modeling in Figures 7C and 7D explains this observation on the basis of nonlinear OFF terminals displaying the steepest luminance tuning curve, and this idea is supported by the results in Figures 8F and 8G: C_1/2_ was lowest (i.e., contrast-sensitivity highest) in nonlinear terminals with Hill coefficients greater than 1.5. Together, the results in Figures 7 and 8 demonstrate how a detailed description of the luminance tuning curve also helps us understand retinal signaling under natural conditions, when the visual stimulus involves fluctuations around a recent mean.

## Discussion

Imaging synaptic vesicle fusion has allowed us to make an in vivo survey of the visual signal as it is transmitted to the inner retina through the population of bipolar cells. Two properties that varied across these synapses affected the transmission of information about the luminance and contrast of a visual stimulus. First, the luminance sensitivities of individual terminals varied across 4 log units, with a log-normal distribution similar to that observed in natural scenes. As a result, the sensitivity of synaptic transmission to a fluctuating stimulus depended on the mean luminance around which this fluctuation occurred relative to the luminance sensitivity of the terminal. Second, about half the synapses employed a triphasic tuning curve in which the largest deflection was a strongly supralinear function of luminance. These unusual tuning curves provided for a high degree of discriminability over a narrow range of luminances and an increased sensitivity to temporal contrast. Triphasic tuning curves also increased the dynamic range over which bipolar cells signal light and improved the efficiency with which luminance information is transmitted: the most efficient terminals used an average of just 1 synaptic vesicle per second per distinguishable gray level.

### Variations in Luminance Tuning across a Population of Synapses

The young fish we used in this study (9–12 dpf) have a retina strongly dominated by cones, reflecting the delayed development of rods (Raymond et al., 1995; Fadool, 2003). Variations in luminance sensitivity are therefore unlikely to reflect mixed rod and cone input. How, then, does this wide variation in luminance sensitivities arise? Bipolar cells are morphologically and functionally diverse (Masland, 2001; Connaughton et al., 2004), and our current understanding of their function suggests a number of possible mechanisms. First, different bipolar cells sum synaptic signals from varying numbers of cones, depending on the size of their dendritic trees. Second, bipolar cells vary in their spectral sensitivities, and the amber stimulus we used in this study will preferentially stimulate red cones. Third, the efficiency with which these synaptic currents spread from dendrites to the synaptic terminal might vary, depending on the resistance of the soma, axon and terminal. Fourth, the change in membrane potential within the synaptic compartment might vary according to the local membrane resistance, either due to variations in the complement of intrinsic conductances, or because of variations in the strength of GABAergic feedback from amacrine cells.

Here, we have measured the intensity-response function and distribution of sensitivities from a dark-adapted state. It will be interesting to assess how coding through the population of synapses alters as the retina adapts to different mean light levels (Rieke and Rudd, 2009). The log-normal distribution of luminance values in natural scenes does not vary between sunrise and sunset (Richards, 1982; Pouli et al., 2010), so it might be predicted that the distribution of synapse sensitivities will be constant in shape but vary in width and shift between different luminance ranges. The relative efficiencies of signaling through ON and OFF channels might then be expected to alter as the mean rate of vesicle release through these two channels change.

### Linear and Nonlinear Synapses

Tuning curves in sensory neurons are usually monotonic (as in photoreceptors encoding luminance; Schnapf et al., 1990) or Gaussian (as in neurons encoding orientation in the visual cortex; Seriès et al., 2004). The triphasic tuning curves observed in about half the bipolar cell terminals were therefore unexpected, but they are consistent with the ERG of primates, where the b-wave, primarily reflecting the response of ON bipolar cells, goes through a maximum termed the “photopic hill” (Ueno et al., 2004).

In many species, it is possible to differentiate linear and nonlinear ganglion cells according to their responses to stimuli varying in time and/or space (Hochstein and Shapley, 1976; Victor et al., 1977). Where do these nonlinearities arise? Cones providing the input to the retinal circuit display relatively simple tuning to luminance: approximately linear for low intensities and then saturating monotonically (Naka and Rushton, 1966b; Normann and Perlman, 1979; Baylor et al., 1987). The next neural compartment in which the visual signal has been recorded is the soma of bipolar cells. Using slices of mouse retina, Euler and Masland (2000) recorded voltage responses in rod bipolar cells and found that the luminance-response curve was linear (Hill coefficient 1.07). Also using mice, Field and Rieke (2002) and Sampath and Rieke (2004) found a weak supralinearity in the light-evoked current recorded in voltage-clamped rod bipolar cells (Hill coefficient 1.5) but no significant nonlinearity in OFF bipolar cells receiving inputs from cones. We have now assayed the visual signal a little further downstream, in the synaptic compartment of the bipolar cell, where we find strong nonlinearities and even switches in signal polarity. The contrast with electrophysiological measurements in mice might be explained by functional differences between mammals and fish, but it may also be that the signal transmitted by bipolar cells is not assessed adequately by measuring electrical signals in the soma.

Neuronal signaling mechanisms consume significant amounts of energy, and the efficient use of spikes and vesicles is one of the constraints affecting the design of neural circuits and the codes they implement (Laughlin, 2001). Here, we have shown that nonlinear synapses encode luminance more efficiently (Figures 7C and 7D) and also have higher sensitivity to contrast (Figure 8). What then is the function of linear terminals? It is hard to answer this question satisfactorily without an overview of how the linear and nonlinear terminals compare in transferring other important properties of a visual stimulus, such as the temporal frequencies it contains. In this study we have only compared how the two populations signal temporal contrast and find that together they allow for detecting changes in contrast over a wide range. The ideal observer model predicts that linear synapses will have lower contrast sensitivities than those with triphasic luminance tuning curves (Figure 7), and experiments demonstrate that linear synapses are capable of signaling changes in contrast when the output of nonlinear synapses approaches saturation (Figures 8D and 8E).

### Nonlinear Synapses: Potential Mechanisms

Although the distinction between synapses that encode luminance linearly and nonlinearly was relatively clear (Figure 5A), we do not know whether this reflects their connections to other neurons in the IPL or a variation in their intrinsic properties. The synaptic terminals of bipolar cells receive direct inhibitory feedback from amacrine cells, many of which have large dendritic trees that integrate signals over a wide area of the retina (Masland, 2001) and which have been shown to feedback onto bipolar cell terminals to control output gain (Zaghloul et al., 2007). Such wide-field amacrine cells might be expected to activate at lower levels of luminance than individual bipolar cells and then generate inhibitory signals that continue to grow as luminance increases and more bipolar cells are activated. Models of the glomerular circuitry in the olfactory bulb suggest that contrast enhancement in mitral cells might occur by a similar mechanism: a local inhibitory interneuron with higher sensitivity, causing the mitral cell to be inhibited at low concentrations of odorant before being stimulated at higher concentrations (Cleland and Sethupathy, 2006).

One source of an intrinsic nonlinearity may be the voltage-dependent calcium channels that control neurotransmitter release, which can generate oscillatory voltage signals and even spikes (Burrone and Lagnado, 1997; Protti et al., 2000; Baden et al., 2011; Dreosti et al., 2011). Variations in the synaptic machinery downstream of the calcium signal, such as the calcium sensor that triggers vesicle fusion, might also exist. For instance, while release from ribbon synapses of rod photoreceptors has a linear dependence on calcium (Thoreson et al., 2004), the most rapid component of release from bipolar cell synapses shows a power law dependence with exponent of 3–4 (Heidelberger et al., 1994; Burrone et al., 2002). Extrinsic factors that might cause variations in tuning curves include the degree of coupling between different terminals (Arai et al., 2010) or inputs from amacrine cells (Baccus, 2007; Gollisch and Meister, 2010). The precise circuit mechanisms that underlie linear and nonlinear transformations of the visual signal are still unclear, but direct visualization of synaptic activity using sypHy or SyGCaMP2 should provide a particularly direct way of testing different models, especially when amacrine cells can also be targeted (Dreosti and Lagnado, 2011).

## Experimental Procedures

### Animals

Zebrafish (*Danio rerio*) were maintained according to Home Office regulations. Fish were maintained as described by Nusslein-Volhard and Dahm (2002) using a 14:10 hr light-dark cycle at 28°C. Fish were kept in E2 medium containing 1-phenyl-2-thiourea (200 μM) from 28 hr postfertilization to minimize pigmentation. Transgenic animals were generated in a mixed genetic background from fish originally purchased from a local aquatic supplier (Scotsdales line), using plasmids taking advantage of the *I-SceI* meganuclease coinjection protocol (Thermes et al., 2002; Supplemental Information). Most imaging was carried out on fish homozygous for the *roy* mutation (Ren et al., 2002) because reduced numbers of iridophores facilitated imaging. SypHy fish on a nonmutant background produced very similar results to those on a *roy* background.

### Multiphoton Imaging In Vivo

Zebrafish (9–12 dpf) were anesthetized by brief immersion in 0.016% Tricaine in E2, immobilized in 2.5% low-melting-point agarose, and placed on a glass coverslip with one eye pointing up. To prevent eye movement after recovering from anesthesia, ocular muscles were paralyzed by nanoliter injections of α-bungarotoxin (2 mg/ml) behind the eye. After mounting in a chamber, fish were superfused with E2. Imaging was carried out using a custom-built two-photon microscope equipped with a mode-locked titanium-sapphire laser (Chameleon, Coherent) tuned to 915–920 nm and an Olympus LUMPlanFI 40× water immersion objective (NA 0.8) (Figure 1A). Emission was captured both by the objective and a substage oil condenser (Olympus, NA = 1.4), through GFP emission filters (HQ 535/50, Chroma Technology) before detection with photomultiplier tubes (Hamamatsu). Laser scanning and image acquisition were controlled using ScanImage v. 3.0 (Pologruto et al., 2003). Light stimuli were generated by an amber LED and 600/10 BP filter and delivered through a light guide placed close to the eye of the fish. Stimulation was synchronized to image acquisition through Igor Pro v. 4.01 software. The mean intensity of the stimulus was controlled by neutral density filters and modulations around this mean by a custom-built LED driver which switched the driving current at 10 kHz while adjusting the duty cycle. The unattenuated stimulus was ∼5.5 × 10^5^ photons/μm^2^/s, and a period of 40 s dark adaptation was interleaved between each presentation of a stimulus. Data were obtained from 42 fish.

### Image Analysis

Movies were analyzed using SARFIA, a custom-written suite of macros running in Igor Pro (Dorostkar et al., 2010). First, movies were registered to correct small lateral movements but were rejected if the plane of focus altered significantly. Next, images were transformed using a Laplace operator and segmented by applying a threshold. The ability of this algorithm to define ROIs corresponding to individual terminals is shown in Figure 3 of Dorostkar et al. (2010). The cross-sectional area of each ROI was measured and the sypHy or SyGCaMP2 signals quantified as the average fluorescence per unit area, after background subtraction. In some terminals, a small linear correction for bleaching was applied, as shown in Figure S2. Terminals were only used for analysis if the response to a step of bright light occurred with a SNR > 4 when imaging at 4 Hz, or SNR > 2 when imaging at 8 Hz. Measurements using sypHy were carried out on a total of 1021 ON and 1995 OFF terminals. Measurements using SyGCaMP2 were carried out on a total of 60 ON and 132 OFF terminals.

Calculations of release rates involved differentiation of the sypHy signal (Equation 1) resulting in an amplification of noise. We therefore calculated the initial rate of release simply by fitting a line to the first 2 or 4 s of the response to a step of light or contrast respectively. For ON terminals, the value of F_min_ for each terminal was calculated in the dark, and for OFF terminals it was calculated over the last 10 s of a 40 s step at ND 1 (see Figure 3D, top graph).

To assess the degree to which the luminance tuning curves were linear, the Hill equation was fit to the relation between luminance and the initial rate of release at light onset. In many terminals, this function was triphasic, so we constrained the fitting procedure to operate between the minimum and maximum of the major deflection of the tuning curve. In a second step, we checked the fits to the Hill function by eye to ensure they gave us reasonable estimates for I_1/2_ and the Hill coefficient.

### Estimating the Rate of Exocytosis from sypHy Signals

To calculate the release rate in a bipolar cell terminal we begin with the following relation:

(Equation 11) $$\frac{{\mathrm{dN}}_{out}}{dt}=V_{exo}\left(t\right)-V_{endo}\left(t\right)$$

where N_out_ is the number of vesicles fused to the terminal membrane and V_exo_ and V_endo_ are the speeds of exocytosis and endocytosis, respectively. Because

(Equation 12) $$V_{endo}\left(t\right)=k_{endo}\cdot N_{out}\left(t\right),$$

the speed of exocytosis is

(Equation 13) $$V_{exo}\left(t\right)=\frac{dN_{out}}{dt}+k_{endo}\cdot N_{out}\left(t\right)$$

where k_endo_ is the rate-constant of endocytosis, which has been measured to be ∼0.1 s^−1^ during ongoing activity in isolated bipolar cells (Neves and Lagnado, 1999) and in vivo (Figure 3B). Fast endocytosis (∼1 s) will not contribute significantly to these estimates because it has a limited capacity and primarily operates on vesicles released within the first tens of milliseconds of a large calcium transient (Neves et al., 2001). Further, the fluorescence of the pHluorin is quenched with a time constant of 4–5 s only *after* endocytosis, reflecting the time required for reacidification of the interior of the vesicle by the proton pump (Granseth et al., 2006). Decay of the sypHy signal with a time constant of 4–5 s was not observed (Figure 3B), consistent with the fast mode of retrieval being very small compared to the much larger number of vesicles retrieved with a time constant of 10 s.

We assume that vesicles are in one of two states; internalized and quenched (with unitary fluorescence, F_vq_), and released and unquenched (F_vu_). A number of studies using pHluorin-based reporters have also demonstrated a standing pool of unquenched reporter on the cell surface (Granseth et al., 2006), so the total sypHy fluorescence F at time t was assumed to be the sum of these three different sources of fluorescence, as follows:

(Equation 14) $$F\left(t\right)=\left(N_{out}\left(t\right)\cdot F_{vu}\right)+\left(\left(N_{total}-N_{out}\left(t\right)\right)\cdot F_{vq}\right)+\left(N_{total}\cdot \alpha_{\min}\cdot F_{vu}\right)$$

where α_min_ is the fraction of vesicles “stuck” on the terminal membrane and not involved in the vesicle cycling process, and N_total_ is the total number of vesicles in the terminal. We estimated α_min_ and N_total_ as described below.

Equation 14 can be arranged to

(Equation 15) $$N_{out}\left(t\right)=\frac{F\left(t\right)-\left(N_{total}\cdot \left(F_{vq}+\left(\alpha_{\min}\cdot F_{vu}\right)\right)\right)}{F_{vu}-F_{vq}}$$

Because F_vq_ = F_vu_/20 (Sankaranarayanan et al., 2000), we can define

(Equation 16) $$N_{out}\left(t\right)=\frac{F\left(t\right)-\left(N_{total}F_{vq}\right)-\left(N_{total}\cdot \alpha_{\min}\cdot 20\cdot F_{vq}\right)}{19\cdot F_{vq}},$$

and as the terminal's minimum fluorescence F_min_ (Figures 3C and 3D) is the sum of the fluorescence of unquenched vesicles stuck on the outside membrane and the remaining vesicles quenched in the cytoplasm of the terminal assuming zero release

(Equation 17) $$F_{\min}=\left(N_{total}\cdot \alpha_{\min}\cdot 20\cdot F_{vq}\right)+\left(N_{total}\cdot \left(1-\alpha_{\min}\right)\cdot F_{vq}\right),$$

we can calculate F_vq_ from

(Equation 18) $$F_{vq}=\frac{F_{\min}}{N_{total}\cdot \left(19\cdot \alpha_{\min}+1\right)}.$$

From Equations 13 and 16 we have

(Equation 19) $$\frac{dN_{out}}{dt}=\frac{dF}{dt}\cdot \frac{1}{19\cdot F_{vq}}$$

which can be differentiated to obtain the relation stated in the main text:

(Equation 20) $$V_{exo}\left(t\right)=a\left[\frac{dF}{dt}+\left(k_{endo}\cdot \left(F\left(t\right)-b\right)\right)\right]$$

where $a=1/19\cdot F_{vq}$ and $b=N_{total}\cdot F_{vq}\cdot \left(1+20\cdot \alpha_{\min}\right).$

### Measurement of the sypHy Surface Fraction, α_min_

In the absence of exocytosis, there are unquenched pHluorin molecules on the surface membrane, equivalent to a fraction α_min_ of all vesicles. This surface fraction can be measured by quenching with acid (Granseth et al., 2006). For ON terminals, the minimal surface fluorescence is reached in the dark, and for OFF terminals, in bright light. These measurements were carried out in intact zebrafish by changing the pH of the bathing medium from 7.4 to 3.2. Averaged measurements are shown in Figure 3C. The surface fraction (α_min_) was then calculated as (ΔF/19.7)/F_pH3.2_. The relative fluorescence of an ON terminal in darkness decreased to 0.84 during acid quenching of surface pHluorin, from which α_min_ = 0.97%. The relative fluorescence of an OFF terminal in bright light decreased to 0.91, from which α_min_ = 0.51%. Because the measuring error in these experiments was high, we used an average value of α_min_ = 0.8% for both ON and OFF terminals. We also estimated α_min_ in dissociated bipolar cells with giant synaptic terminals using epifluorescence microscopy, as shown in Figure S3. The value we obtained α_min_ = 1.7% was somewhat higher than the value we obtained in vivo.

### Estimating the Total Vesicle Pool (N_total_)

The average density of vesicles in a bipolar cell terminal was calculated as ∼1050 per μm^3^ using electron micrographs of retinal slices 80 nm thick (Schmitt and Dowling, 1999; Figure 3A). N_total_ was then calculated for each terminal by multiplying the density by the volume of the terminal (T_Vol_). T_Vol_ was not measured by full 3D reconstruction of each terminal, but by assuming that the optical section we were imaging contained the center of the terminal, which was shaped spherically. To minimize errors in this estimate, the thickness of the optical section was increased to ∼2.5 μm by reducing the numerical aperture of the IR beam used for multiphoton imaging. The average diameter of a bipolar cell terminal in these images was about 3 μm, which is very similar to estimates made from electron micrographs.

## Supplemental Information

Document S1. Figures S1–S6, Table S1, and Supplemental Experimental Procedures

Movie S1. Synaptic Vesicle Fusion Imaged in the Retina In Vivo The upper panel shows raw fluorescence in the IPL speeded up 50 times, and the lower panel shows the corresponding “difference movie” (obtained by subtracting a “baseline” image averaged over the first 20 s from all frames). The orange arrow points in the direction of the photoreceptors. Periods of light stimulation are indicated with an orange dot in the upper right corner, and the numerals inside the dots indicate the ND filter used. This movie is part of the experiment illustrated in Figures 2A–2D (180 to 520 s). Terminals become brighter when synaptic vesicle fusion accelerates and dimmer when vesicle fusion becomes slower than retrieval by endocytosis. In the difference movie, medium gray represents no change. Inner Nuclear Layer (INL); Inner Plexiform Layer (IPL); Ganglion Cell Layer (GCL).
