# Supplementary material for: Encoding of Luminance and Contrast by Linear and Nonlinear Synapses in the Retina
Source: Neuron. 2012 Feb 23;73(4-2):758–73. doi: 10.1016/j.neuron.2011.12.023 (PMC3314971; doi:10.1016/j.neuron.2011.12.023)
Supplement: Document S1. Figures S1–S6, Table S1, and Supplemental Experimental Procedures [file mmc1.pdf]

Neuron, Volume *73*

Supplemental Information

Encoding of Luminance and Contrast by Linear and Nonlinear Synapses in the Retina

Benjamin Odermatt, Anton Nikolaev, and Leon Lagnado

Inventory:

Supplemental information includes Figure S1 showing immune-fluorescence staining (addition to Figure 1). Figure S2 showing initial responses of the fluorescent reporters to the imaging laser and examples of bleach correction (addition to Figures 2E and F and Figure 6G). Figure S3 showing α_{min} calculations on dissociated bipolar cells (addition to Figure 3C). Figure S4 showing distributions of the Hill coefficient and $I_{1/2}$ from SyGCaMP2 experiments (addition to Figure 5). Figure S5 showing tuning curves of individual example terminals for sypHy and SyGCaMP2 (addition to Figure 6). Figure S6 showing additional measurements of contrast-sensitivity as a function of luminance (addition to Figure 8). Supplemental information also includes a movie of the example experiment in Figures 2A-D as well as additional molecular biology information, including Table S1 with primers used for PCR cloning, and a detailed immunohistochemistry protocol.

Supplementary Figure 1

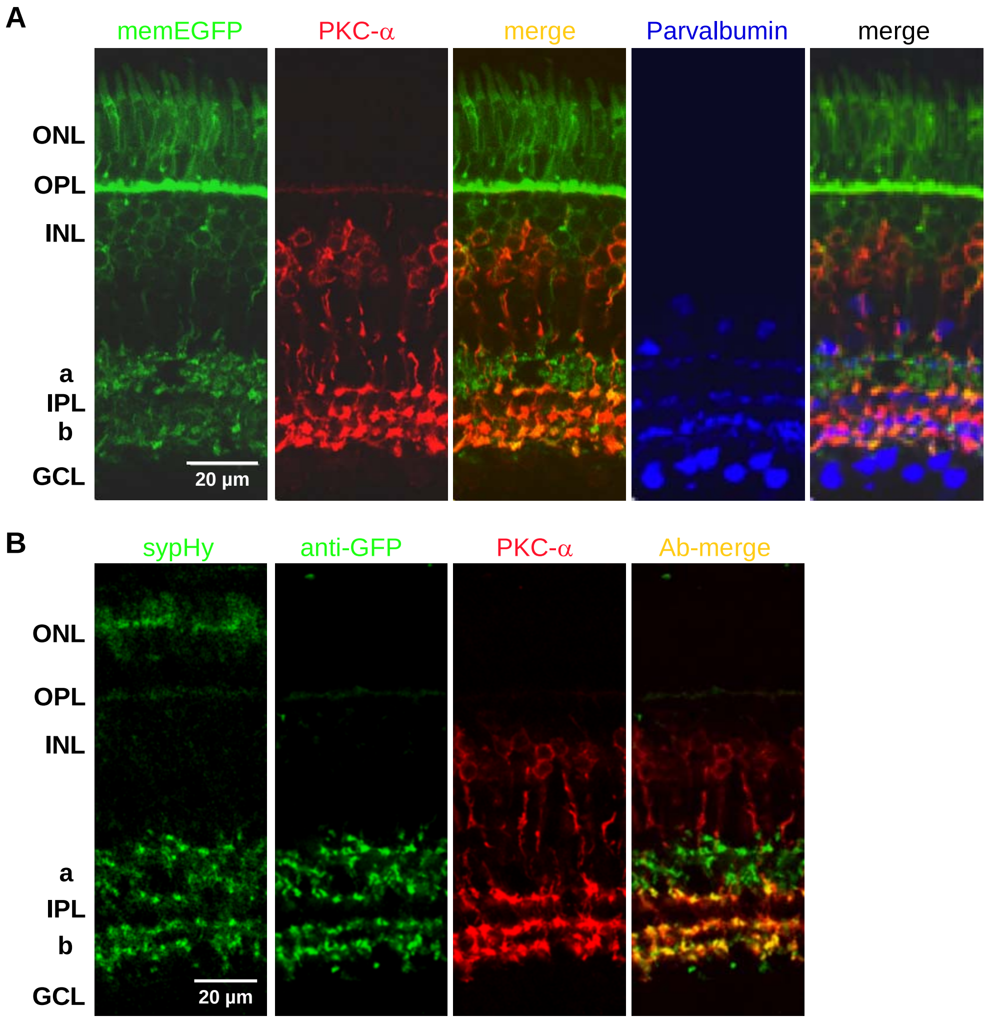

Odermatt et al.

Supplementary Figure 2

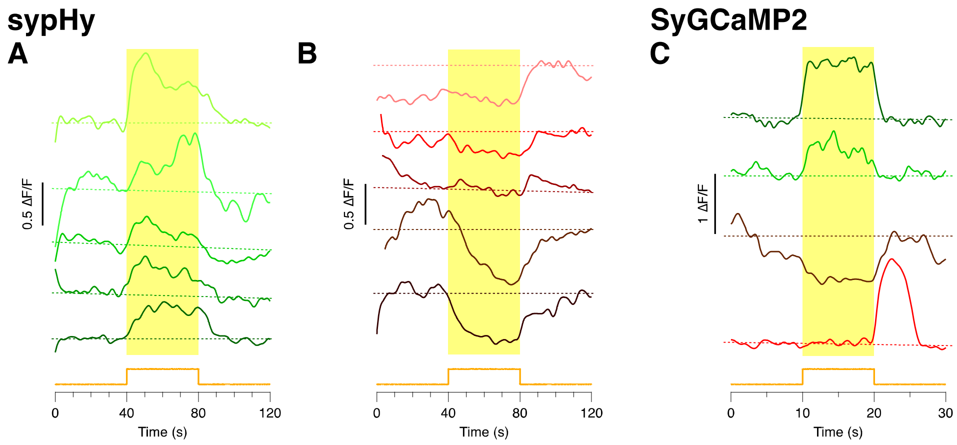

Supplementary Figure 3

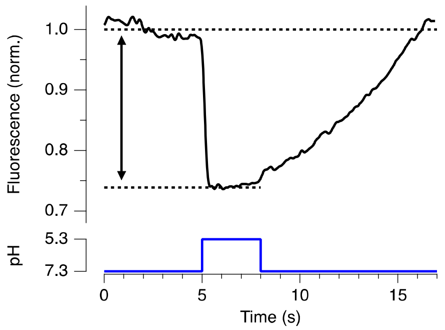

Odermatt et al.

Supplementary Figure 4

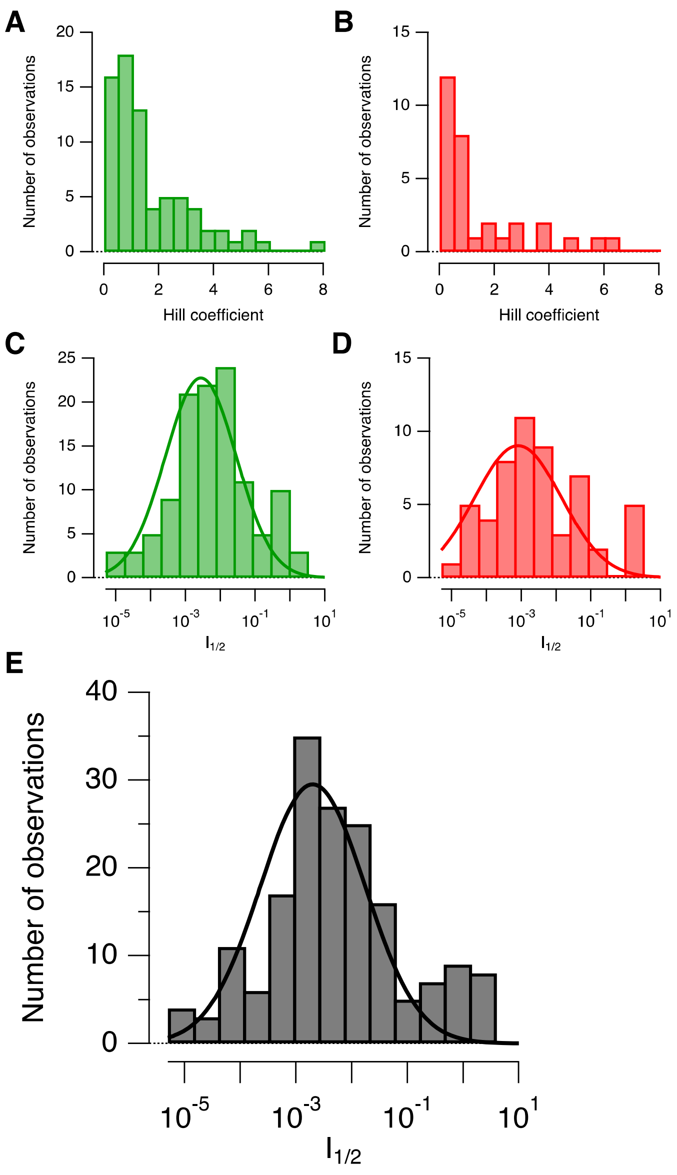

Odermatt et al.

Supplementary Figure 5

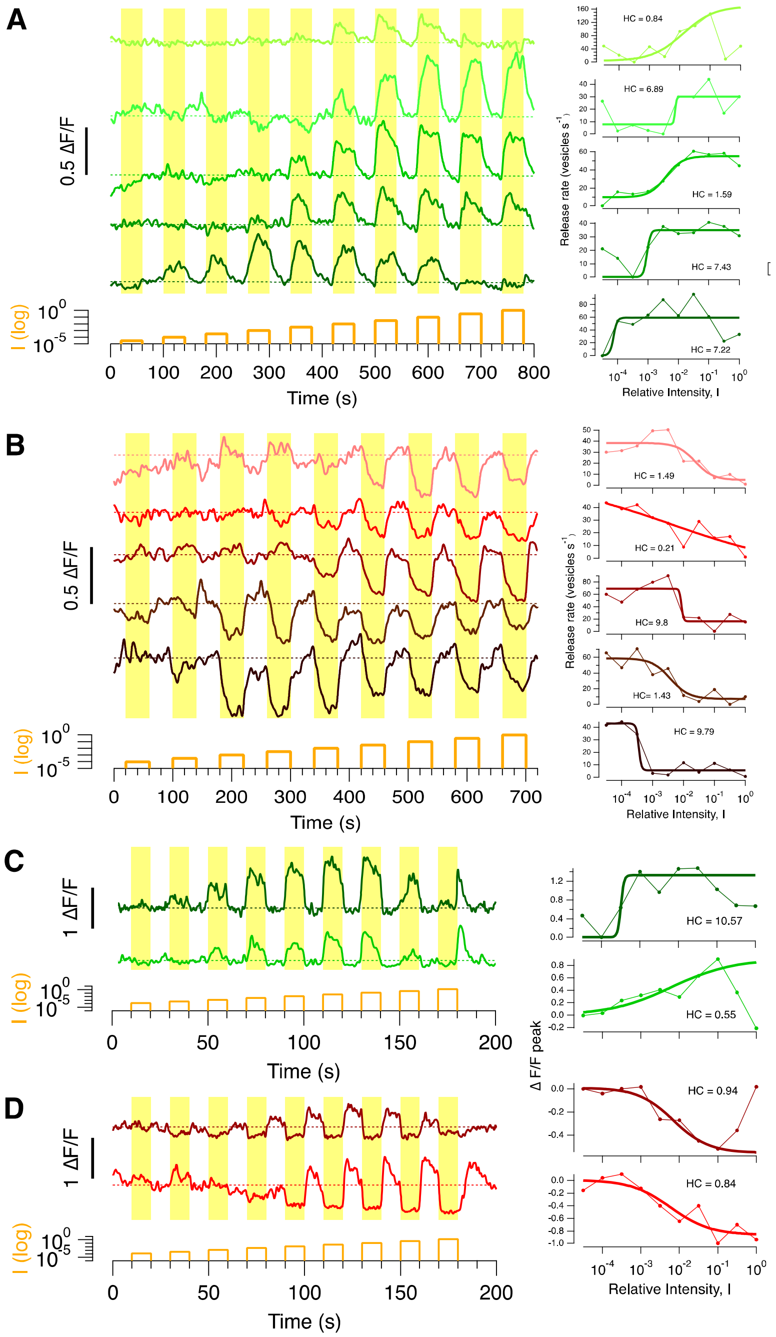

Odermatt et al.

Supplementary Figure 6

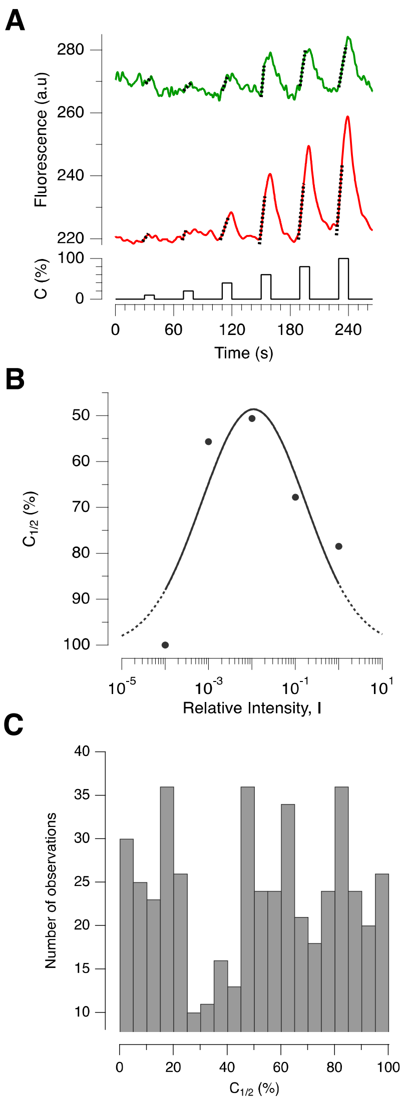

Odermatt et al.

Supplemental Figure Legends

Figure S1. Expression of fluorescent proteins in the zebrafish retina under the *ribeye a (ctbp2)* promoter

(A) Confocal images of a retinal section from a stable transgenic line of fish (*Tg(-1.8ctbp2:memEGFP)lmb)* expressing membrane-targetted EGFP under the ribeye a promoter (8 dpf). The image shows a densely packed layer of photoreceptors in the outer nuclear layer (ONL), their terminals in the outer plexiform layer (OPL), cell bodies of bipolar cells in the inner nuclear layer (INL) and their terminals in the inner plexiform layer (IPL, sublamina a and b). Note the complete overlap between memEGFP and ON bipolar cells positive for PKCα staining (red). The neurons stained blue are positive for parvalbumin, a marker of A2 amacrine cells and displaced amacrine cells in the ganglion cell layer (GCL): note that the ribeye a promoter does not drive expression in these (merged image to right). (B) Confocal images of a retinal section from a stable transgenic line of fish (*Tg(-1.8ctbp2:sypHy)lmb)* expressing sypHy under the ribeye a promoter (10 dpf). SypHy expression has been marked with an anti-GFP antibody. Expression is driven in photoreceptor terminals in the outer plexiform layer (OPL), and in bipolar cell terminals in the inner plexiform layer (IPL, sublamina a and b). There was complete overlap of sypHy expression and anti-PKCα staining (red, ON-bipolar terminals) in sublamina b of the IPL, while PKCα negative terminals were mainly situated in sublamina a (OFF-bipolar terminals). There is also green auto-fluorescence in photoreceptor outer segments.

Figure S2. Initial responses of sypHy and SyGCaMP2 to the imaging laser and linear bleach correction

(A) Examples of the sypHy signal when scanning of the IR laser is initiated (ON terminals). These traces are from the examples in Figure 2E and Supplementary Figure 5A. Responses of varying magnitude are seen, but all adapt to the IR laser within 5-10 s. A full-field step of light (relative intensity 10^{-1}) was applied at 40 s. Dotted lines through the prestimulus baselines were used for linear bleach corrections. (B) Same as in (A) but for OFF terminals shown in Figure 2F and Supplementary Figure 5B. (C) Examples of the SyGCaMP2 signal when scanning of the IR laser is initiated (two ON terminals above and two OFF terminals below).

These traces are from the examples in Figure 6G and Supplementary Figures 5C and D. Because of the better SNR provided by SyGCaMP2, the intensity of the scanning laser was typically about half that used to image sypHy, so in most cases there was very little response to the imaging laser and little bleaching.

Figure S3. Estimation of the sypHy surface fraction (α_{min}) in dissociated bipolar cell terminals

Epifluorescence measurements of α_{min} were carried out on bipolar cells dissociated from the retina of adult sypHy fish (similar as described in (Lagnado et al., 1996)). These are Mb cells with giant synaptic terminals receiving mixed rod and cone inputs. The measurements were made in 0 Ca^{2+} medium to ensure absence of spontaneous vesicle release. The trace is the average quenching of fluorescence F on application of a quenching solution at pH 5.3 (n = 5 cells). The total fluorescence decreased by 25%, equivalent to α_{min} = 1.7% (calculated as $\alpha_{min} = (\Delta F/19.7)/F_{pH5.3}$, as described in the Experimental Procedures).

Figure S4. Non-linearities and sensitivity across the population of bipolar cell synapses measured with SyGCaMP2

Distribution of Hill coefficients (h) and sensitivities $I_{1/2}$ measured with SyGCaMP2 in ON terminals (green) and OFF terminals (red). The response to a step of light was measured as the initial rate of change of SyGCaMP2 fluorescence relative to resting fluorescence. Imaging rate = 8 Hz. ON terminals (green, n = 132) are shown in (A) and (C), OFF terminals (red, n = 60) are shown in (B) and (D). Similar to the sypHy measurements both distributions in (A) and (B) show a distinct population with $h < 1.5$ (termed "linear"), and a second population with $h > 2.0$ ("non-linear"). Distribution of sensitivities $I_{1/2}$ measured with SyGCaMP2 across a complete sample of ON terminals (green) are shown in (C) and OFF (red) in (D). The fitted curves are log-normal functions. (E) Distribution of $I_{1/2}$ across the complete sample of ON and OFF synapses together (n = 192). I_0, the peak of the distribution, occurs at a relative intensity of $2.0 \pm 0.6 \times 10^{-3}$, which is not significantly different from the value of $1.47 \pm 0.39 \times 10^{-3}$ measured with sypHy. The width of the distribution $\sqrt{2}\sigma$ was 3.06 ± 0.41 log units.

Figure S5. Examples of luminance tuning curves in individual terminals

(A) Left: sypHy measurements in ON terminals (as in Figure 2E). Right: corresponding luminance tuning curves. Continuous lines are fits of the Hill equation applied to the largest amplitude change. The Hill coefficient (HC) is shown next to each trace. (B) SypHy measurements of luminance tuning in OFF terminals (as in Figure 2F). (C) Left: SyGCaMP2 measurements in ON terminals (as in Figure 6G). Right: corresponding luminance tuning curves. Continuous lines are fits of the Hill equation applied to the largest amplitude change. (D) SyGCaMP2 measurements in OFF terminals (as in Figure 6G). For each tuning curve the Hill coefficient (HC) of the corresponding Hill fitting (bold traces) is given.

Figure S6. Distributions of contrast sensitivities $C_{1/2}$

(A) Larger scale view of averaged responses of ON (green; n = 200) and OFF (red; n = 340) terminals to contrasts varying between 10% and 100% at a relative mean intensity of 10^{-2} (corresponding to ~5.5 x 10^3 photons/μm^2/s). Black dotted lines are linear fits over the first 4 seconds of each contrast response to demonstrate how vesicle release has been calculated. (B) The contrast generating a half-maximal exocytic response ($C_{1/2}$) plotted as a function of the mean luminance. Results are averaged across all ON and OFF terminals. The fitted curve is a log-normal function, with peak at I = 1.09 ± 0.73 x 10^{-2} and width $\sqrt{2}\sigma$ of 3.89 ± 0.95 log units. Note the similarity with the distribution of luminance sensitivities shown in Figure 5C. (C) The distribution of contrast sensitivities, $C_{1/2}$, averaged across ON and OFF terminals. For each terminal, $C_{1/2}$ was measured at a mean luminance within 0.5 log units of the luminance at which $C_{1/2}$ was maximal. Note the two peaks in the distribution: one population of terminals with high contrast sensitivities ($C_{1/2} < 25$ %) and a second population with low contrast sensitivities ($C_{1/2} > 45\%$). These two peaks are likely to reflect the populations of terminals with linear and non-linear tuning curves, for which the ideal observer model in Figures 7C and D predicts different contrast sensitivities.

Supplemental Experimental Procedures

Molecular Biology

For the generation of reporter plasmids containing the *ribeye a* promoter, we first cloned a backbone I-SceI injection plasmid. The pBluescript® II KS+ phagemid (Stratagene) was used in which we flanked the existing multi-cloning side with two additional *I-SceI* recognition sites by PCR cloning into the *BssHII* sites using primers I-SceI forward and I-SceI reverse (see Table 1). Then 1.8 kb of the zebrafish *ribeye a (ctbp2)* promoter upstream of the ATG start codon was PCR cloned from genomic zebrafish DNA using the primers ribeye_a forward and reverse. The primers, comprising a PmeI and NheI restriction site, respectively, were designed from the chromosome 17 genomic sequence contig NW_633757.1 (GI:67045643) to amplify the sequence region from bp 907289 to 905464 (zebrafish *ribeye a* ATG at 905453). This zebrafish *ribeye a* promoter was then ligated into the AleI and XbaI sites of the I-SceI pBluescript® plasmid.

To target EGFP to the surface membrane (memEGFP), a fusion was made with the N-terminal palmitoylation domain of neuromodulin (GAP-43), a neurospecific calmodulin-binding protein, which has been shown to effectively transport and anchor EGFP to the membrane (Liu et al., 1994). A double-stranded oligonucleotide, fused from mem-Sens and mem-Antisense primers (see Table 1), was cloned into the AgeI site of the commercially available pEGFP-N1 vector (BD Biosciences, Clontech). This oligonucleotide contained the DNA sequence for the N-terminal 20 amino acids of this palmitoylation domain, as well as AgeI overhanging ends and a BglII recognition sites

To generate the I-SceI *ribeye a (ctbp2)*:mem-EGFP injection plasmid, the sequence of memEGFP followed by a SV40 polyA domain from BamHI to SspI was cloned into the BamHI and EcoRV sites of the I-SceI *ribeye a* promoter plasmid upstream of the promoter. To generate the I-SceI *ribeye a (ctbp2)*:sypHy injection plasmid the rat-sypHy sequence (Granseth et al., 2006) followed by a SV40 polyA domain from NheI to SspI was cloned into the SpeI and EcoRV sites of the I-SceI *ribeye a* promoter plasmid.

Table S1.

Primer name:	Primer sequence 5' to 3':
I-SceI forward	ccc gcg cgc tag gga taa cag ggt aat tgg gta ccg ggc ccc ccc tcg
I-SceI reverse	ggg gcg cgc att acc ctg tta tcc cta aag gga aca aaa gct gga gct cca ccg

ribeye_a forward	aac gtt taa act tgg aat tat taa aaa agg c
ribeye_a reverse	gga gct agc tat acc tta ctc aca ggg aag aaa g
Mem-Sens	ccg gtc gcc acc atg ctg tgc tgt atg aga aga acc aaa cag gtt gaa aag aat gat gag gac caa aag atc tta
Mem-Antisense	ccg gta aga tct ttt ggt cct cat cat tct ttt caa cct gtt tgg ttc ttc tca tac agc aca gca tgg tgg cga

Immunohistochemistry

Transgenic zebrafish larvae 8 to 10 dpf were anesthetized in 0.05% MS222 (Tricaine methanesulfonate; Sigma) and fixed in 4% paraformaldehyde in PBS (in mM: 1.5 NaH_2PO_4, 8 Na_2HPO_4, and 145 NaCl, pH 7.3) for 30 min at room temperature (RT). The fish were then cryoprotected in 30% sucrose in PBS at 4°C over night. 30 µm horizontal whole mount cryostat sections were then permeabilized using 0.1% Triton X-100 in PBS for 10 min at RT. Non-specific sites were blocked using PBS containing 4% normal goat serum and 3% bovine serum albumin (blocking buffer). Antibodies were diluted to their final concentration in blocking buffer. Primary antibodies (anti-PKCα, Sigma P4334, 1:2000; anti-Parvalbumin, Chemicon MAB1572, 1:500; anti-GFP, Chemicon MAB3580, 1:500) were applied for 2 hr at RT. Cuts were rinsed once in blocking buffer and three times for 15 min with PBS, and then appropriate fluorophore-tagged secondary antibodies (Alexa fluor®-conjugated goat anti-mouse or –rabbit, Invitrogen, 1:1000) were applied for 2 hr at RT. Finally, cuts were washed five times for 15 min with PBS and mounted onto slides with PermaFluor™ aqueous mounting medium (Thermo Scientific). Confocal images with an optical thickness of 2.5 µm were taken on a Nikon Eclipse E800 upright microscope equipped with a BIO-RAD Radiance 2010 (plus) confocal unit.

Supplemental References

Granseth, B., Odermatt, B., Royle, S.J., and Lagnado, L. (2006). Clathrin-mediated endocytosis is the dominant mechanism of vesicle retrieval at hippocampal synapses. Neuron *51*, 773-786.

Lagnado, L., Gomis, A., and Job, C. (1996). Continuous vesicle cycling in the synaptic terminal of retinal bipolar cells. Neuron *17*, 957-967.

Liu, Y., Fisher, D.A., and Storm, D.R. (1994). Intracellular sorting of neuromodulin (GAP-43) mutants modified in the membrane targeting domain. J Neurosci *14*, 5807-5817.
