# Supplementary figures and images for: Encoding of Luminance and Contrast by Linear and Nonlinear Synapses in the Retina
Source: Neuron. 2012 Feb 23;73(4-2):758–73. doi: 10.1016/j.neuron.2011.12.023 (PMC3314971; doi:10.1016/j.neuron.2011.12.023)

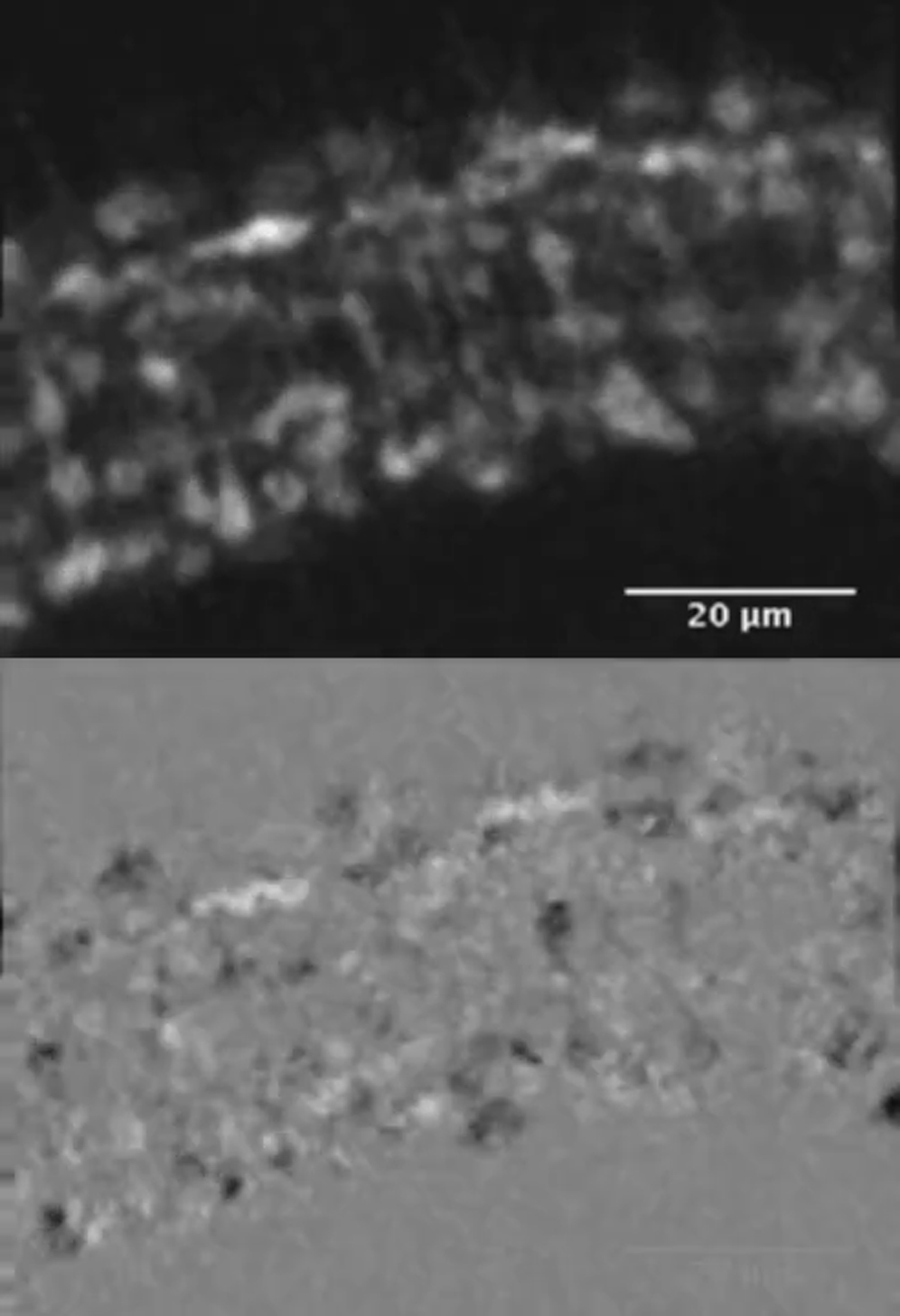

Supplement: Movie S1. Synaptic Vesicle Fusion Imaged in the Retina In Vivo — The upper panel shows raw fluorescence in the IPL speeded up 50 times, and the lower panel shows the corresponding “difference movie” (obtained by subtracting a “baseline” image averaged over the first 20 s from all frames). The orange arrow points in the direction of the photoreceptors. Periods of light stimulation are indicated with an orange dot in the upper right corner, and the numerals inside the dots indicate the ND filter used. This movie is part of the experiment illustrated in Figures 2A–2D (180 to 520 s). Terminals become brighter when synaptic vesicle fusion accelerates and dimmer when vesicle fusion becomes slower than retrieval by endocytosis. In the difference movie, medium gray represents no change. Inner Nuclear Layer (INL); Inner Plexiform Layer (IPL); Ganglion Cell Layer (GCL). [file mmc2.jpg]
